# ﻿A new species of *Halorhynchus* from Madagascar (Coleoptera, Curculionidae, Cossoninae, Onycholipini)

**DOI:** 10.3897/zookeys.1100.75987

**Published:** 2022-05-16

**Authors:** Maria Lourdes Chamorro, Warren Steiner

**Affiliations:** 1 Systematic Entomology Laboratory, Agricultural Research Service, USDA c/o Smithsonian Institution, Washington, DC, USA Agricultural Research Service, USDA c/o Smithsonian Institution Washington United States of America; 2 Research Collaborator, Smithsonian Institution, Department of Entomology, NHB 187, Washington, DC, 20013, USA Smithsonian Institution, Department of Entomology, Washington United States of America

**Keywords:** Africa, Australia, overseas dispersal, psammophilous, weevils

## Abstract

*Halorhynchusremii* Chamorro & Steiner, **sp. nov.** is described from Madagascar. This new species is the third known species of the genus and the first for Africa. *Halorhynchusremii* is compared to other psammophilous, anophthalmous onycholipine cossonines. Transoceanic dispersal between Australia and Madagascar and sand burrowing adaptation are briefly discussed. A key to the species is provided.

## ﻿Introduction

**Figure 1.**
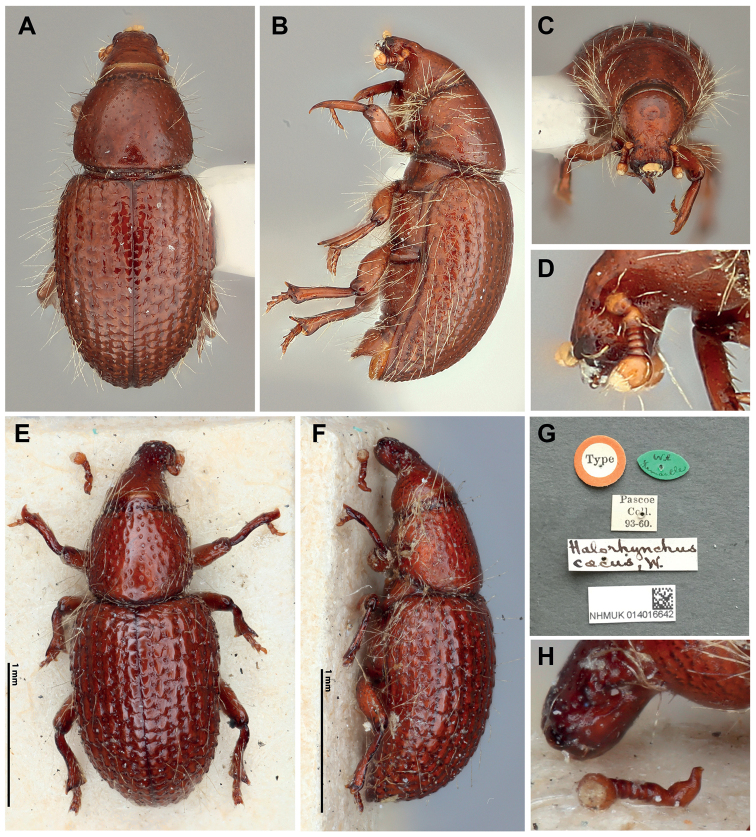
**A–D***Halorhynchusremii*, new species, holotype **A** dorsal view **B** lateral view **C** anterior view **D** detail of antenna, lateral view **E–H***Halorhynchuscaecus*, NHMUK014016642, type **E** dorsal view **F** lateral view **G** labels **H** detail of antenna, lateral view.

*Halorhynchus* Wollaston, 1873, is a cossonine genus known from two species in Western Australia: *Halorhynchuscaecus* Wollaston, 1873 (Fig. 1E–H) and *H.geniculatus* Lea, 1900. The genus was originally described in Pentarthrini by Wollaston (1873) based on the presence of a five-segmented funicle, parallel body outline, absence of a scutellum, slight separation between each of the fore- and midlegs, as well as what Wollaston considered to be pentarthrid-like abdominal segments and tibia. Alonso-Zarazaga and Lyal (1999) suitably transferred the genus to Onycholipini, a tribe containing at least four genera that are also fossorial, anophthalmous, and/or psammophilous (Morrone and Hlavac 2017): *Dipnotyphlus* Colonnelli from Yemen, *Hauseriola* Osella, 1980 from Crete, *Leipommata* Wollaston, 1857 (Fig. 3A–D) from Madeira, Selvagens, the Canary Islands and Morocco, and *Onycholips* Wollaston, 1861 (Fig. 3E–H) from the Canary Islands and Morocco. Wollaston (1861) remarked on the resemblance between *Halorhynchus* and the Madeiran *Onycholips*, and contemplated describing *Halorhynchuscaecus*, an Australian species, in *Onycholips* (Wollaston 1873: 527).

*Halorhynchus* differs from the four above mentioned genera as follows: the five-segmented funicle sets *Halorhynchus* apart from *Onycholips*, which has six funicular segments; *Leipommata* has seven funicular segments. *Dipnotyphlus* and *Hauseriola* have five-funicular segments as *Halorhynchus*, but the scape is widened distally in *Dipnotyphlus*. In addition, species in *Hauseriola* and *Dipnotyphlus* have the rostrum almost twice longer than wide, the elytra are subapically laterally constricted in *Dipnotyphlus* and both genera bear large, deep dorsal punctation and lack the elongate, stramineous hairs present in both *Onycholips* and *Halorhynchus*. In addition, *Dipnotyphlus* and *Hauseriola* are pseudotetramerous and have the third tarsomere bilobed.

On the sandy shores of southwestern Madagascar, the second author (WS) dug around the roots of sprawling vegetation, under natural beach debris of twigs and seaweed and hand collected, during the day, a single anophthalmous cossonine weevil with psammophilous features. Since 1993 the specimen has remained identified only to family-level in the National Weevil Collection (National Museum of Natural History, Smithsonian Institution, Washington, D.C., USNM).

During an ongoing study of the weevils of Madagascar, the first author (LCh) was made aware of this weevil. Initially, the specimen appeared to represent a new genus, as research into possible African anophthalmous, psammophilous cossonines did not yield any results. However, in 2019, while studying Wollaston’s collection at the Natural History Museum, London (NHMUK) LCh determined this species to belong to *Halorhynchus*, a cossonine genus, known until now only from Western Australia. In the results we indicate the characters that support this determination.

Generally, cossonine weevils prefer dead and decomposing plant tissue, usually boring and scarring recently felled trees (Jordal 2014; Pierce 1918); some, such as *Caulophilus* Wollaston (Kuschel 1962; Pierce 1918) and *Dynatopechus* Marshall (Zimmerman and Anderson 1949), are serious pests of grains and beans, respectively. Some cossonines in Dryotribini, Neumatorini, Onycholipini, and Pentarthrini are endogean, dwelling deep in soil and humus (Morrone and Hlavac 2017), while others in Rhyncolini, Proecini, and some Onycholipini create complex galleries in wood involving adult and larval tunnels and egg-niches, similar to those created by scolytine bark and platypodine ambrosia beetles (Kuschel 1966; Osella 1982; Kato 1998; Mecke and Galileo 2004; Jordal 2014). Finally, the newly transferred *Phylloplatypus* Kato (Kuschel et al. 2000; Jordal et al. 2011) feeds on and mines the living leaf tissue between the veins of *Pandanus* Parkinson and are able to transmit fungal pathogens from infected to uninfected leaves (Sugiura and Masuya 2010), the spores of which may be carried by the adult in mycangia located on the forecoxae (Morimoto and Kojima 2004).

Not much is known about the biology of *Halorhynchus*. Lea (1900) collected *Halorhynchusgeniculatus* in Geraldton, Western Australia while these weevils were burrowing at the roots of a small saltbush (*Atriplex* sp., Amaranthaceae) in the ‘outer beach’, which we assume corresponds to the back shore or coastal sand dune area. *Halorhynchuscaecus* was collected by Lea (1900) on the beach near the roots of spinifex grasses, *Spinifex* sp. (Poaceae).

To date, the only subterranean cossonine weevils recorded from Madagascar are *Pentebathmusinsularis* Richard and *P.ovalis* Richard (Dryotribini) (Remillet 1973; Morrone and Hlavac 2017). Here we describe the third known species of *Halorhynchus* and the first for the African region.

## ﻿Materials and methods

Habitus photographs were captured with the Macropod Pro (Microscopic Solutions). Individual images were taken at various focal planes and combined with Zerene Stacker (Zerene Systems, LLC). Plates were created and labeled with a combination of Adobe Photoshop and InDesign (Adobe Products). The map of the distribution of *Halorhynchus* was created using SimpleMappr (Shorthouse 2010). The holotype specimen is deposited in the National Museum of Natural History (**USNM**) in Washington, D.C. As part of the permit agreement reached with the Malagasy government, holotypes were to be kept at the USNM with identified duplicates, if any, at the University of Madagascar Collection. No duplicates exist.

## ﻿Results

The following characteristics suggested inclusion of this species in *Halorhynchus*, until now, known only from Western Australia: presence of a five-segmented funicle, club glabrous with apical tomentose area circular and reduced, rostrum approximately as long as wide, pronounced longitudinal constriction between frons and base of rostrum, parallel body outline, absence of a scutellum, elytral striae forming regular rows and punctation distinct, interstriae moderately raised, weakly serrate, body reddish-brown, shiny, with elongate stramineous lateral setae, eyes absent, legs moderately-sized, femora unarmed; tibiae apically expanded, with strong, curved uncus on outer, anterior (dorsal) margin, hind tibiae fossorial; tarsi with 4 subequal tarsomeres, linear, fourth tarsomere absent, fifth tarsomere apically acute with 2 distal setae; tarsal claws obsolete [applies to only fore- and midlegs since holotype of *Halorhynchuscaecus* is missing the apical tarsomeres of the hind leg (see Fig. 1E, F)].

### ﻿ Halorhynchus remii

Taxon classificationAnimaliaColeopteraCurculionidae

Chamorro & Steiner sp. nov.

B0294001-1F19-5E66-AFDC-637E7436EEC8

http://zoobank.org/6AAFA8DA-C19F-44AF-9E88-1640E1C714AA

Figs 1A–D , 2A , 3B

#### Material examined.

***Holotype***: USNMENT00896694 Madagascar: Prov. Toliara; Ifaty 23°09'S, 43°37'E, 18 September 1993 // under leaf litter beneath spreading plants on dry dunes; coll. W.E. Steiner, R. Andriamasimanana (USNM).

#### Diagnosis.

The new species strongly resembles the Western Australian species *Halorhynchuscaecus*, but can be distinguished from it by the pronotum being wider 2/3 from the base, the shallower and relatively smaller pronotal punctation, the more acutely pointed apex (uncus) of the foretibiae, the hind tarsi in lateral view with setose, elongate, sclerotized tarsal extensions, and clawlessness.

#### Description.

Size: 2.0 mm. Reddish-brown, oval, shiny, with elongate stramineous lateral setae on body. ***Head***: short, not basally constricted. Frons convex, foveola reduced. Eyes absent. Rostrum as long as wide (dorsal and lateral view), narrower than frons and head, widening apically, dorsally with weak longitudinal depression at base of rostrum; apex truncate. Scrobes short, reaching base of rostrum, directed ventrad, expanding proximally, shallow. Antennae short, located approximately one-third from base of rostrum; scape short, almost subequal to first funicular segment; funicle 5-segmented, segment 1 equal to 2 and 3 combined, segments 2–5 dorso-ventrally compressed, subequal size and shape; club short, elliptical, as long as segments 2–5 combined, shiny, appearing 1-segmented, apex with clump of medially directed elongate, stout, stramineous setae. Hypostomal tooth pronounced. Proventriculus not dissected. ***Thorax***: Prothorax trapezoidal with rounded anterior and posterior angles, widest at basal third, narrowed anteriorly but not compressed or rimmed; punctation sparse, shallow, small. Scutellum absent. Elytra oval; with 9 well-marked striae, interstriae slightly raised, rugous almost serrate, toothed apicad (posterad) and laterad humeri weak. Hind wing not dissected. Forecoxae narrowly separated, distance less than tarsal width, mid-coxae slightly more separate than forecoxae, hind coxae widely separated, distance twice greatest coxal diameter. Mesanepisternal suture distinct, mesanepisternum broadly deltoid, widest anterad, mesepimeral suture distinct, mesepimeron small, deltoid, approximately a quarter the size of mesanepisternum. Metasternum three times wider than long, femoral impression absent; sclerolepidia present; metanepisternum narrow, elongate, almost four times longer than wide; metanepisternal suture becoming obscure posterad; venter sparsely and shallowly punctate, apically. ***Legs***: moderately-sized, femora unarmed; tibiae apically expanded, with strong, curved uncus on outer, anterior (dorsal) margin, foretibia bearing a row of stramineous setae, uncus of mid- and hind tibiae shorter and more blunt, hind tibiae fossorial; tarsi with 4 subequal tarsomeres, linear, fourth tarsomere absent, fifth tarsomere apically acute with 2 distal setae (homology assessment based on the reduction of the fourth tarsomere in most Curculionoidea, however there are Curculionoidea that have five visible tarsomeres, a secondary elongation of 4^th^ tarsomere, as in *Dryophthorus* Schönherr and *Stenommatus* Wollaston); tarsal claws obsolete, hind tarsi in lateral view with setose, elongate, sclerotized tarsal extensions (Fig. 1B). ***Abdomen***: ventrites 1 and 2 without visible suture, apparently fused, ventrites 3–5 as long as 1 and 2 combined. Single specimen not dissected.

#### Etymology.

The species is named in honor of the late Rémy Lemagnen, who was devoted to the study of weevils on the island of Reunion. The specific epithet also suggests the Latin word for oar (*remus*) given the weevil’s sand burrowing and ‘rowing’ habit.

#### Biology.

*Halorhynchusgeniculatus* was reported from saltbush (*Atriplex* sp.) from Geraldton, Western Australia (Lea 1900). *Atriplex* spp are suffruticose coastal species that have a woody base but apical parts that die off after flowering. Lea (1900) also indicated he found *H.caecus* on the beach near the roots of spinifex grasses approximately 4 inches below the surface. *Atriplexperrieri* Leandri is the only species of the genus known to occur on Madagascar, on the coastal areas of the southern part of the country (Sukhorukov 2013). Given several assumptions, *Halorhynchusremii* may be found to be associated with *Atriplexperrieri* or other coastal adapted plant species.

### ﻿Key to the known species of *Halorhynchus*

*Halorhynchusgeniculatus* was not examined and the key is based on the description and differential diagnosis provided by Lea (1900).

**Table:** 

1	Pronotum wider 2/3 from base; punctation shallow and small (distance between punctation more than twice diameter of each puncture); uncus apically narrowed and pointed; tarsomeres ventrally with digitate projection (Fig. 1B); claws obsolete	***Halorhynchusremii* sp. nov.**
–	Pronotum widest medially; punctation deeper and larger (distance between punctation between 1.5–1.0 diameter of each); uncus apically broad with a small point; tarsomeres ventrally without digitate projection (Fig. 1F); claws simple, thin	**2**
2	Abdomen distinctly punctate; size 2 mm, width 4/5 mm or less (additional non-discrete characters provided by Lea (1900) are smaller, thinner, paler, shorter setae, and the anterior uncus shorter and thinner)	***Halorhynchusgeniculatus***
–	Abdomen not as distinctly punctate; size greater than 2 mm long and 4/5 mm wide (larger, wider, darker, setae longer, and anterior uncus longer and wider)	***Halorhynchuscaecus***

## ﻿Discussion

The ability of certain insects, such as cossonine weevils, to disperse long distances overseas supports our placement of this new species in an already described genus previously only known from Australia, almost 9000 km from Madagascar. We consider it unlikely and less parsimonious that the Australian and Malagasy species belong to two different lineages. The numerous shared external characters we observe (as listed above) of the species is probably not due to convergent evolution as a result of adaptation to similar niche space.

The body form of *Halorhynchusremii* and other psammophilous weevils have converged in other beetle families with similar habits, as in some species of burrowing Tenebrionidae: *Phaleria* Latreille, *Trachyscelis* Latreille and *Chaerodes* White, as well as several ultra-psammophilous tenebrionids in the Molurini and Tentyriini (Koch 1962), as well as the Scarabaeidae*Leiopsammodius* Rakovic (Harpootlian et al. 2000) and saprinine histerids (Lackner et al. 2019). These all differ from the sand surface dwellers and “sand swimmers”, which have long slender legs.

Burrowing beetles such as the ones mentioned above remain underground for most of their life where they may be feeding on roots, and only emerging to find possible mates. These beetles are often found at the interface between dry and damp sand. The adaptation of psammophilous beetles, with their compact body, stout antennae and legs, elongate stiff setae (mostly on ventral surfaces) may aid in locomotion through sand (Koch 1962). The elongate hairs assist in preventing abrasion by keeping sand grains away from joints (Koch 1962). The front tibiae are modified for digging and are often toothed or spatulate. The expanded apex of the middle and hind tibiae often bear stout, peg-like or spatulate setae and spurs for pushing through sand. Tarsi are usually short, reduced, and capable of folding back along the tibia or fitting into tibial grooves. Body color is often pale due to a depigmentation of the cuticle and the eyes reduced or absent, similar to cave species. Most psammophilous species are flightless (Koch 1962; Carpaneto and Fattorini 2002). The new species shares all of these features.

Several weevils, especially cossonines, are masters at long-distance overseas dispersal and are able to colonize even the most remote areas on the planet (Zimmerman 1938c; 1938e; 1940; Decelle and Voss 1972; Thompson 1989; Cristóvão and Lyal 2018). Many weevils, as adults and larvae, are endophytic in dead or dying wood, including cossonines. These weevils can tolerate submersion in saline waters for extended periods and have been collected from driftwood, groynes, and structural timbers on coastal habitats of the Atlantic, Indian, and Pacific Oceans (Zimmerman 1940; Hyman and Parsons 1992; Oevering et al. 2000; Kojima and Yoshitake 2020). For example, the onycholipine *Pselactusspadix* (Herbst) can tolerate immersion within a saline environment for up to 7 hours per day (Sawyer and Cragg 1995). This species was found to be widespread and common in coastal regions of England and Wales inhabiting old structural timber even in intertidal zones, which remain submerged during extended periods of times (Oevering et al. 2000). In North America, *Elassoptesmarinus* Horn (Cossoninae) is found on the beach and is associated with driftwood.

**Figure 2.**
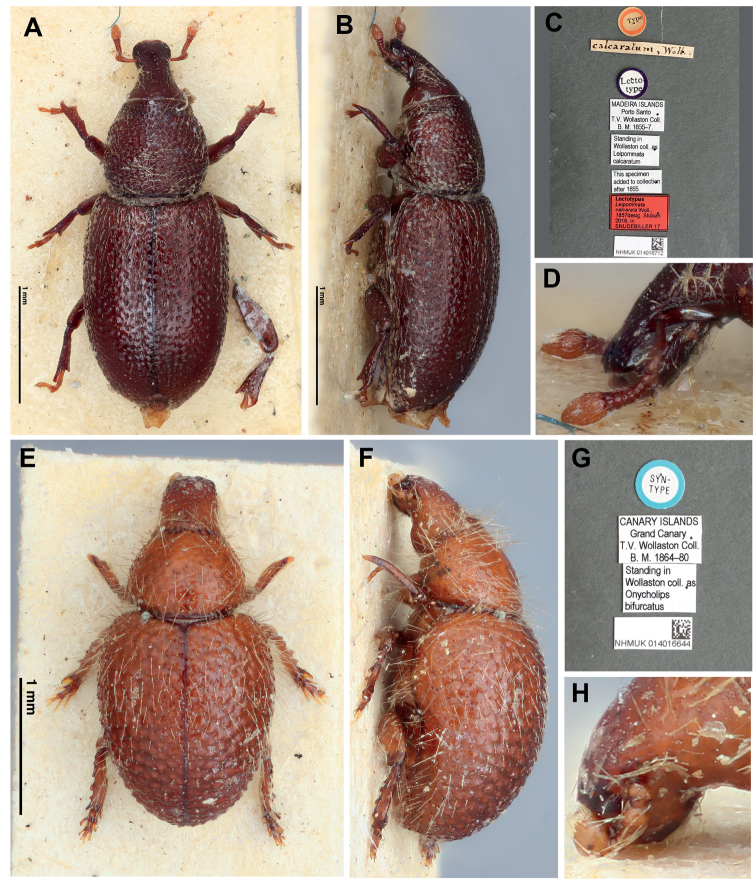
**A–D***Leipommatacalcarata*, NHMUK014016712, lectotype **A** dorsal view **B** lateral view **C** labels **D** detail of antenna, lateral view **E–H***Onycholipsbifurcata*, NHMUK014016644, syntype **E** dorsal view **F** lateral view **G** labels **H** detail of antenna, lateral view.

No other species best illustrates this capability to disperse overseas than the widely distributed *Dryotribusmimeticus* Champion, which probably drifts on warm oceanic surface currents (Fig. 2C). This species has been reported from localities very distant from one another: the Seychelles in the Indian Ocean; Honshu, Japan; Alligator Bay and Adele Island, Australia; remote islands and atolls, including Wake, Hawaii, Johnston Island, and the Galapagos Islands in the Pacific; coastlines bordering the Caribbean Sea and Gulf of Mexico in the North Atlantic (Zimmerman 1940; O’Brien and Wibmer 1982; Alonso-Zarazaga et al. 2017). The likelihood that humans transported this species to these areas by way of hitchhiking in ancient ship ballasts filled with surface beach sand (Lindroth 1957; 1968; Steiner 2002) is low given the remoteness of some of these places. Rather, this species has probably dispersed while rafting on ocean currents within drifting debris (Zimmerman 1942; Howden 1977; Solomon 2004).

Several examples of marine dispersal have been suggested for beetles: a survey of the beetles on a beach in New South Wales, Australia, revealed greater affinities between beetles found on beach debris with that of Pacific island faunas than with the nearby forest (Howden 1977); divergence-time estimation of Malagasy Scolytinae and the timing of major planetary geological events suggest overseas dispersal for several lineages on the island (Jordal 2013); non-native weevils have washed ashore and colonized remote islands in Japan (Kojima and Yoshitake 2020). Furthermore, transoceanic dispersal on marine drift includes reports of an overseas 16,000 km journey from South America to Australia of a single log (Barber et al. 1959), some of which transport endophytic organisms such as termites (Schuchert 1935); entire landmasses washing off and drifting to sea (Darlington 1957); live ants washing ashore in Santa Clara Island (San Sebastian), Spain from Brazil (Wheeler 1916), summarized in Holzapfel and Harrell (1968).

We consider the cossonines to be a rather complicated group of weevils in that they are not only highly speciose, with more than 1700 species described (Jordal 2014), but compared to other organisms, including other members of the superfamily Curculionoidea, appear to disperse relatively effortlessly across vast oceanic distances and tolerate the harsh oceanic environment.

All known cossonines are endophytic (Jordal 2014) and this new species is probably no exception. Lea (1900) indicated *Halorhynchusgeniculatus* to be found near the root system of an Australian *Atriplex* sp. and he collected *H.caecus* near the roots of *Spinifex* sp. grasses. *Halorhynchusremii* may also be associated with the roots of certain plants, including *Atriplex* sp. in Madagascar. While the above examples of cossonine marine dispersal deal with species that live inside timber, such as *Pselactus*, species of *Halorhynchus* may have dispersed within the root system of their host plants. A plausible scenario would be co-dispersal within or with the root system of its host plant. Regarding directionality of the dispersal, this will require additional study, however, a possible scenario may have been as follows assuming *Atriplex* is a host plant of *Halorhynchus*: *Atriplex* colonized Australia twice independently during the late Miocene once from Central Asia (6.3–4.8 Mya) and once from Eurasia or America (9.8–7.8 Mya) (Kadereit et al. 2010). *Halorhynchus* has never been included in a phylogenetic analysis, however, as cossonines originated in the mid-Cenozoic (McKenna et al. 2009) the possibility of co-dispersal during the late Miocene from areas in Central Asia, Eurasia, or America to Australia cannot be discredited. Thompson (1989) was able to hypothesize directionality of transoceanic dispersal (New Zealand to Chile) by comparing the morphology of known species of *Euophryum*. He found the northern New Zealand species to resemble most closely the Chilean species than the southern New Zealand species and also a lack of variability in the spermatheca of the Chilean species throughout its range suggested a more recent introduction (Thompson 1989). However, this type of comparison is not yet possible with species of *Halorhynchus* due to low number of individuals known.

**Figure 3.**
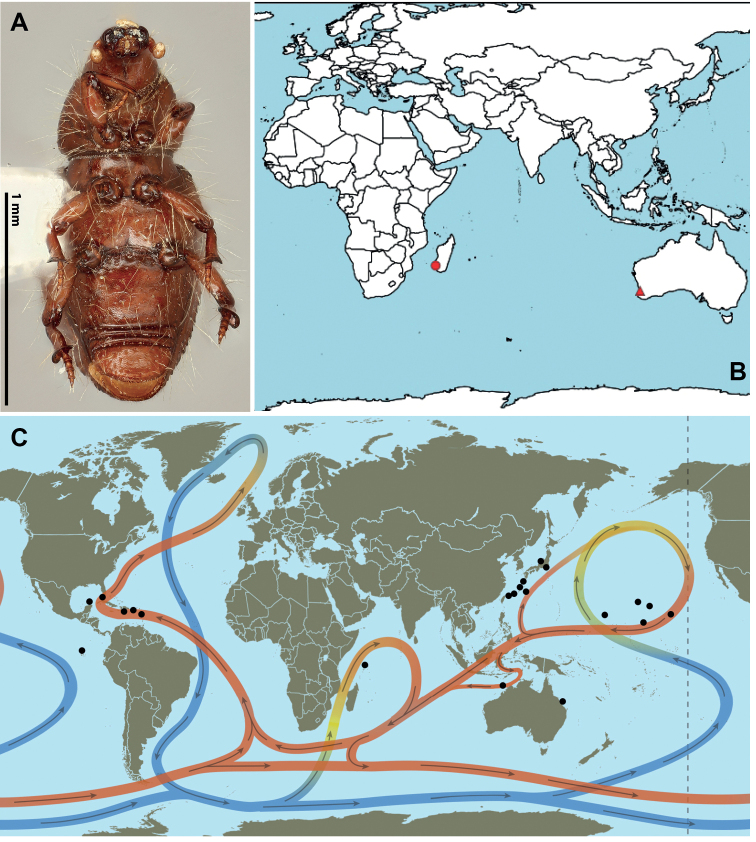
**A***Halorhynchusremii*, holotype, ventral view **B** Distribution of *Halorhynchus* species: circle: *Halorhynchusremii*; triangle: *Halorhynchuscaecus* and *H.geniculatus***C** distribution of *Dryotribusmimeticus* and ocean currents; red lines depict warm surface currents, blue are deeper, cold currents (sources Intergovernental Panel on Climate Change via Smithsonian magazine; rendition by Taina Litwak, USDA ARS SEL).

Cossonine classification and assessment of higher-level groups requires a global perspective. This subfamily presents a unique challenge due to the ability of its members to readily disperse, colonize, and radiate, which hints to potentially high levels of taxonomic synonymy in the group.

We hope that this research sheds light on the ability of these cossonine weevils to disperse long distances and informs future work on the group as new species and genera continue to be discovered and described. We also hope this stimulates research on coastal areas where insects are rarely surveyed; particularly in areas in the African continent. Discovery of *Halorhynchus* on other parts of Africa may continue to shed light on the dispersal of this and other groups of cossonine weevils.

## Supplementary Material

XML Treatment for Halorhynchus remii
